# We need both natural and energy solutions to stabilize our climate

**DOI:** 10.1111/gcb.14612

**Published:** 2019-03-22

**Authors:** Bronson W. Griscom, Guy Lomax, Timm Kroeger, Joseph E. Fargione, Justin Adams, Lucy Almond, Deborah Bossio, Susan C. Cook‐Patton, Peter W. Ellis, Christina M. Kennedy, Joseph Kiesecker

**Affiliations:** ^1^ The Nature Conservancy Arlington Virginia; ^2^ Tropical Forest Alliance, World Economic Forum Geneva Switzerland

**Keywords:** climate change, fossil fuel emissions, land use, natural climate solutions, terrestrial ecosystems

## Abstract

We respond to concerns raised by Baldocchi and Penuelas who question the potential for ecosystems to provide carbon sinks and storage, and conclude that we should focus on decarbonizing our energy systems. While we agree with many of their concerns, we arrive at a different conclusion: we need strong action to advance both clean energy solutions and natural climate solutions (NCS) if we are to stabilize warming well below 2°C. Cost‐effective NCS can deliver 11.3 PgCO_2_e yr^‐1^ or ~30% of near‐term climate mitigation needs through protection, improved management, and restoration of ecosystems, as we increase overall ambition.

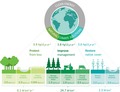

In a recently published opinion piece, Baldocchi and Penuelas (2019) caution against relying on ecosystems as a major climate mitigation opportunity, counter to our findings (Griscom et al., 2017). Interestingly, we agree with many of Baldocchi and Penuelas' concerns, yet we arrive at a different conclusion.

Most fundamentally, we debate the question posed by Baldocchi and Penuelas “if it is more feasible to decarbonize our energy system and reduce carbon emissions, rather than rely on ecosystems [to] take up carbon in a slow, incremental way over current baseline?” This is a false dichotomy. The IPCC (2018) and most recently Anderson et al. (2019) conclude that the world needs aggressive actions to reduce fossil fuel emissions and pursue land‐based options to stabilize warming well below 2°C. Land‐based options include natural climate solutions (NCS) which use ecosystems for removal and storage, and off‐site storage using options like bioenergy with carbon capture and storage (BECCS). Baldocchi and Penuelas suggest that political capital and resources are insufficient to effectively pursue both fossil fuel emissions reductions and NCS. We are pleased to note that climate policies are already successfully integrating both in places like California (https://www.arb.ca.gov/cc/pillars/pillars.htm#pillars) and New Zealand (https://www.mfe.govt.nz/node/23439), and NCS is prominent in nationally determined contributions to the Paris Climate Agreement (Grassi et al., 2017). More options allow greater overall ambition by reducing total cost to society for any abatement goal, given the wide range of marginal abatement costs of potential carbon sequestration strategies (Aldy, Krupnick, Newell, Parry, & Pizer, 2010). Furthermore, ecosystem‐based options have been disproportionately underinvested in relative to their cost‐effective climate mitigation potential (Buchner, Trabacchi, Mazza, Abramskiehn, & Wang, 2015), not to mention co‐benefits like water filtration, flood control, and biodiversity habitat. While we agree that ecosystems should not be considered an alternative to decarbonizing the energy system, they are nonetheless essential to addressing climate change.

We agree with Baldocchi and Penuelas that one must account for albedo as well as saturation of ecosystem sinks as we have done (Griscom et al., 2017). For example, we excluded reforestation in boreal regions due to albedo. We find that saturation will begin in 20–30 years for two of the 20 NCS we analyzed. Most other options are effective beyond 2,100. In any case, saturation is not a concern during the critical first half of this century when we must balance carbon emissions with removals.

We also agree with Baldocchi and Penuelas’ concerns about the permanence of terrestrial ecosystem carbon storage in the face of climate change. Yet, ignoring opportunities to increase terrestrial sinks through NCS would increase rather than avoid this risk. Achieving cost‐effective NCS would provide about 30% of near‐term climate mitigation needs—and hence reduce climate risks—while increasing resilience to climate change and adding only 1% to terrestrial carbon storage that is exposed to climate risks (Griscom et al., 2017). Baldocchi and Penuelas emphasize small carbon fluxes per m^2^ of land. Yet, summed to the globe, gross ecosystem carbon fluxes are an order of magnitude larger than anthropogenic emissions (Denman et al., 2007). While ecosystem fluxes are nearly balanced, net terrestrial sinks absorb one‐fourth of anthropogenic emissions, and could absorb considerably more with NCS. Terrestrial ecosystems also store four times more carbon than the atmosphere (Le Quere et al., 2018). Given the large role ecosystems already play in global carbon fluxes and stocks, the threat of climate change is a further reason to restore resilient ecosystems, rather than a reason to disregard them.

The points raised by Baldocchi and Penuelas about competition for land are also important. They state that “Much land is not available or is unsuitable because it is already dedicated to providing food and fiber for a burgeoning world population.” They report our estimate that “48 M km^2^ are needed with a portfolio of reforestation, avoided forest conversion, forest and crop management, and peat restoration (Griscom et al., 2017)” which they suggest is unrealistic. We agree that this extent is unrealistic, as it is the “maximum with safeguards” extent we report for NCS. The *needed* NCS mitigation potential is about half of this extent (27.1 M km^2^) that can cost‐effectively deliver about 30% of additional terrestrial mitigation in 2030 (11.3 PgCO_2_e yr^‐1^), assuming less mature carbon storage options like BECCS are not yet available (Field & Mach, 2017). Only 9% (2.3 M km^2^) of this needed extent would displace existing land use, primarily by restoring degraded grazing lands with native forest, productive plantations, and agroforestry. The remaining 24.8 M km^2^ involves improving management on working forests and farms while maintaining or increasing long‐term food and fiber yields, and a relatively small extent of avoided loss of forests, wetlands, and grasslands (see graphical abstract online, derived from numbers in Griscom et al., 2017). Nevertheless, we share Baldocchi and Penuelas’ fundamental concern that the major changes we call for in global land use and management must be done with careful consideration of the consequences for food and fiber, not to mention alignment with renewable energy (Kiesecker & Naugle, 2017).

Improving global land stewardship and largely eliminating fossil fuel emissions are both massive undertakings. Nevertheless, both can and must be done in the coming decades to avoid greater costs to society posed by climate change. Done together in smart ways, we can grow our economies and improve our quality of life while stabilizing our climate. Indeed, it is now an imperative of self‐interest that we protect and restore life on earth at an unprecedented scale.

## CONFLICT OF INTEREST

The authors listed above have no conflict of interest to declare.
